# Growth of Carbon Nanocoils by Porous α-Fe_2_O_3_/SnO_2_ Catalyst and Its Buckypaper for High Efficient Adsorption

**DOI:** 10.1007/s40820-019-0365-y

**Published:** 2020-01-16

**Authors:** Yongpeng Zhao, Jianzhen Wang, Hui Huang, Tianze Cong, Shuaitao Yang, Huan Chen, Jiaqi Qin, Muhammad Usman, Zeng Fan, Lujun Pan

**Affiliations:** 1 School of Physics, Dalian University of Technology, Dalian, 116024 Liaoning People’s Republic of China; 2 School of Microelectronics, Dalian University of Technology, Dalian, 116024 Liaoning People’s Republic of China; 3 State Key Laboratory of Fine Chemicals, School of Chemical Engineering, Dalian University of Technology, Dalian, 116024 Liaoning People’s Republic of China; 4 Department of Physics, Khawaja Fareed University of Engineering and Information Technology, Rahim Yar Khan, 64200 Pakistan

**Keywords:** Carbon nanocoils, Porous α-Fe_2_O_3_/SnO_2_, Catalyst, Buckypaper, Methylene blue adsorption

## Abstract

High-purity (99%) carbon nanocoils (CNCs) have been synthesized by using porous α-Fe_2_O_3_/SnO_2_ catalyst. The yield of CNCs reaches 9,098% after a 6 h growth. This value is much higher than the previously reported data, indicating that this method is promising to synthesize high-purity CNCs on a large scale. It is considered that an appropriate proportion of Fe and Sn, proper particle size distribution, and a loose-porous aggregate structure of the catalyst are the key points to the high-purity growth of CNCs. Benefiting from the high-purity preparation, a CNC Buckypaper was successfully prepared and the electrical, mechanical, and electrochemical properties were investigated comprehensively. Furthermore, as one of the practical applications, the CNC Buckypaper was successfully utilized as an efficient adsorbent for the removal of methylene blue dye from wastewater with an adsorption efficiency of 90.9%. This study provides a facile and economical route for preparing high-purity CNCs, which is suitable for large-quantity production. Furthermore, the fabrication of macroscopic CNC Buckypaper provides promising alternative of adsorbent or other practical applications.

**Electronic supplementary material:**

The online version of this article (10.1007/s40820-019-0365-y) contains supplementary material, which is available to authorized users.

## Highlights

- High-purity (~ 99%) carbon nanocoils (CNCs) without the amorphous carbon layer were synthesized by using porous α-Fe_2_O_3_/SnO_2_ catalyst.
- The highest yield of the CNCs can reach ~ 9098% after a 6 h growth, which is much higher than those mentioned in previous reports.
- A CNC Buckypaper was successfully prepared and utilized as an efficient adsorbent for the removal of methylene blue dye with the adsorption efficiency of 90.9%.

**Electronic supplementary material:**

The online version of this article (10.1007/s40820-019-0365-y) contains supplementary material, which is available to authorized users.

## Graphical abstract

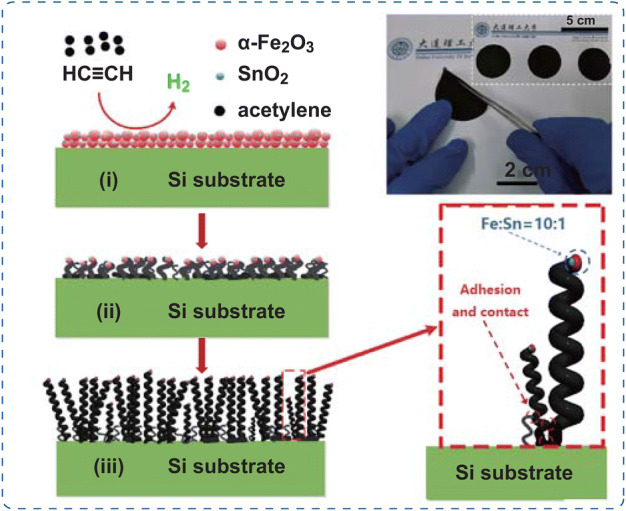

## Introduction

Carbon nanocoils (CNCs), one of the distinctive types of carbon nanomaterials, have attracted wide interests due to their unique helical morphology and attractive properties. Owing to their inherent properties, CNCs hold many potential applications in a wide range of technologies, such as micro-mechanical units [1, 2], strain sensors [3, 4], electromagnetic wave absorbers [5–10], electromagnetic wave shielding [11], field-emission displays [12, 13], nanoactuators [14, 15], supercapacitors [16–20], anodes for lithium ion batteries [21], and nanocomposite photocatalyst [22]. To achieve these applications, large-scale, low-cost, and high-purity production methods are essential.

Catalytic chemical vapor deposition (CVD) method is widely used to synthesize CNCs because of its controllable reaction process, economical cost, and convenient for industrial large-scale production. In this method, selection of appropriate catalysts is crucial for synthesis of CNCs. Therefore, diversified types of catalysts, including Fe [23, 24], Co [25], Ni [26, 27], Cu [28], and multi-component alloys catalysts such as Fe/Sn [29–31], Fe/Sn/In [32, 33], K/Au [34], K/Ag [35], BaSrTiO_3_/Sn [36], Na/K [37], Ni/P [38], and TiC [39] have been investigated for growth of CNCs. Although some improvements were made in raising the purity and yield of CNCs using different systems of catalysts, the low CNC purity is still a challenging issue. The main problem is that the high-purity CNCs are mainly present on the surface of carbon deposits, and there is always an amorphous carbon layer with a thickness ranging from several to tens of microns between the CNC layer and substrate [40–42]. This amorphous carbon layer mixed in the products seriously reduces the purity of CNCs and introduces additional problems of purification. The main reason for this problem is considered to be that the proportion of catalyst particles suitable for the growth of CNCs is not high in the whole input catalysts. In addition, the density and morphology of the initial state catalysts on the substrate are also the key points for the growth of CNCs. In order to overcome this problem, some valuable work has been performed, Hirahara et al. successfully improved the growth efficiency of CNCs by introducing an extra SnO_2_ buffer layer between the catalyst layer and substrate, the thickness of by-product carbon layer was reduced by 50%, and the growth rate was improved 200% compared with the substrate without coating SnO_2_ [41]. Takehiro et al. reduced the thickness of by-product carbon layer to 1/3 by designing a patterned catalyst thin film based on the principle of suppressing catalyst collision [42]. However, the use of lithography or magnetron sputtering technology does not make it possible for large-scale industrial production of CNCs. In any case, a facile and low-cost approach to achieve high-purity CNCs is a crucial but unsettled issue. On the other hand, the production of macroscopical freestanding Buckypapers by using carbon nanomaterials, such as carbon nanotube [43], graphene [44, 45], and carbon nanofiber [46] as building blocks, becomes an important step toward their potential applications. Therefore, the successful preparation of CNC Buckypaper is a marker for CNCs to be synthesized in high purity with large quantity.

The porous α-Fe_2_O_3_/SnO_2_ catalyst shows excellent ability to synthesize CNCs with high efficiency, and it can be easily prepared by a one-pot solvothermal method with low-cost precursor. By using this catalyst in a CVD process, high-purity CNCs were synthesized, without the amorphous carbon layer and the yield of 9098% was achieved after a 6 h growth. Based on the experimental results, the growth mechanism of synthesizing high-purity CNC was investigated. Benefiting from the high-purity and efficient preparation, a CNC Buckypaper was prepared for the first time and the electrical, mechanical, and electrochemical properties were investigated. Finally, as one of the practical applications, the CNC Buckypaper was successfully utilized as an efficient adsorbent for the removal of methylene blue dye.

## Experimental Methods

### Preparation of Porous α-Fe_2_O_3_/SnO_2_ Catalyst

In a typical experiment, 0.05 mmol soluble Fe^3+^ salt was dissolved in N, N-dimethylformamide (DMF); then, a certain amount of soluble Sn^4+^ salt with a molar ratio of Fe^3+^ to Sn^4+^ from 1:0 to 3:1 were added in the solution correspondingly. After ultrasonication for 30 min, the mixture was transferred into a 100-mL Teflon-lined stainless autoclave and heated at 180 °C for 30 h. After reaction, the autoclave was cooled to room temperature naturally. The generated catalyst powder was collected by vacuum filtration using the cellulose membrane with pore size of 0.22 μm, washed with deionized (DI) water and absolute ethanol for three times, and finally dried at 60 °C for 3 h.

### Synthesis of High-Purity CNCs

The catalyst powder (20 mg) was dispersed into 20 mL absolute ethanol. After ultrasonication for 30 min, 50 μL catalyst dispersions were spin-coated on a Si substrate (size: 15 × 15 mm^2^) with a rotation speed of 2000 rpm for 30 s and dried at 40 °C for 10 min. By repeating the spin-coating process, the catalyst films with different densities were obtained. CNCs were produced on these substrates using an atmospheric pressure CVD system at 710 °C for 30 min by introducing a mixture of 235 sccm Ar and 25 sccm C_2_H_2_ gases. During heating and cooling processes, the CVD system was flushed with 250 sccm Ar and the schematic of CVD apparatus with substrate position is shown in Fig. S1. The purity of CNCs is given by Eq. 1:

1 $${\text{Purity}}_{\text{CNC}} = \frac{{N_{\text{total}} - N_{\text{CNF}} }}{{N_{\text{total}} }} \times 100\%$$

where *N*_total_ is the number of all CNCs and carbon nanofibers (CNFs), and *N*_CNF_ is the number of CNFs on the substrate. The number of the CNCs and CNFs was quantified by observing the SEM images of the top-view and cross-sectional SEM images. Furthermore, CNFs with spring-like, twist-like, or braided-like structure were defined as CNCs.

The yield of CNCs is calculated by Eq. 2:

2 $${\text{Yield}}_{\text{CNC}} = \frac{{M_{\text{total}} - M_{\text{Catalyst}} }}{{M_{\text{Catalyst}} }} \times 100\%$$

where *M*_total_ is the total mass of CVD product, and *M*_Catalyst_ is the mass of the catalyst.

### Fabrication of CNC Buckypaper

The as-grown CNCs (200 mg) were removed from the substrates and dispersed in 100 mL nitric acid (68 wt%) at 60 °C for 2 h. This was followed by washing the suspension several times with DI water. After that, 50 mg acid-treated CNCs were dispersed in DI water (100 mL) and treated by ultrasonication in a bath sonicator for 30 min. Then, the CNC dispersions were poured onto a cellulose membrane with pore size of 0.22 μm and filtrated by a vacuum filtration setup. After filtration, the filter paper was dried in an oven at 60 °C for 24 h, and then, a freestanding CNC Buckypaper was peeled off from the filter membrane. The schematic of fabrication process is shown in Fig. S2.

### Characterization

The morphologies of products were characterized using a field-emission scanning electron microscope (FE-SEM, NOVA NanoSEM 450) and a transmission electron microscope (TEM, JEOL JEM-2100). Energy-dispersive X-ray spectroscopy (EDX), high-resolution transmission electron microscopy (HRTEM), and element mapping of the samples were also carried out. X-ray photoelectron spectroscopy (XPS, VG ESCALAB 250Xi), X-ray diffraction (XRD, PANalytical BV Empyrean), Raman spectroscopy (Renishaw in via plus, 532.8 nm laser excitation) were used to characterize the chemical compositions and structures of the samples. The Brunauer–Emmett–Teller (BET) surface area measurement was recorded at 77 K (QUADRASORB SI-KR/MP, Quantachrome, USA). The mechanical property of the CNC Buckypaper characterized by a tensile machine Yl-S370, and the electrical property was monitored using an Agilent Technologies B2902A. The electrochemical measurements of the CNC paper were carried out using a CHI660E electrochemical workstation. Adsorption characteristics of methylene blue on CNC Buckypaper and CNC powder were measured by using a UV–Vis spectrophotometer (PerkinElmer, Lambda 750 s).

## Results and Discussion

### Growth of High-Purity CNCs

#### Effects of Molar Ratios of Fe and Sn

**Fig. 1:**
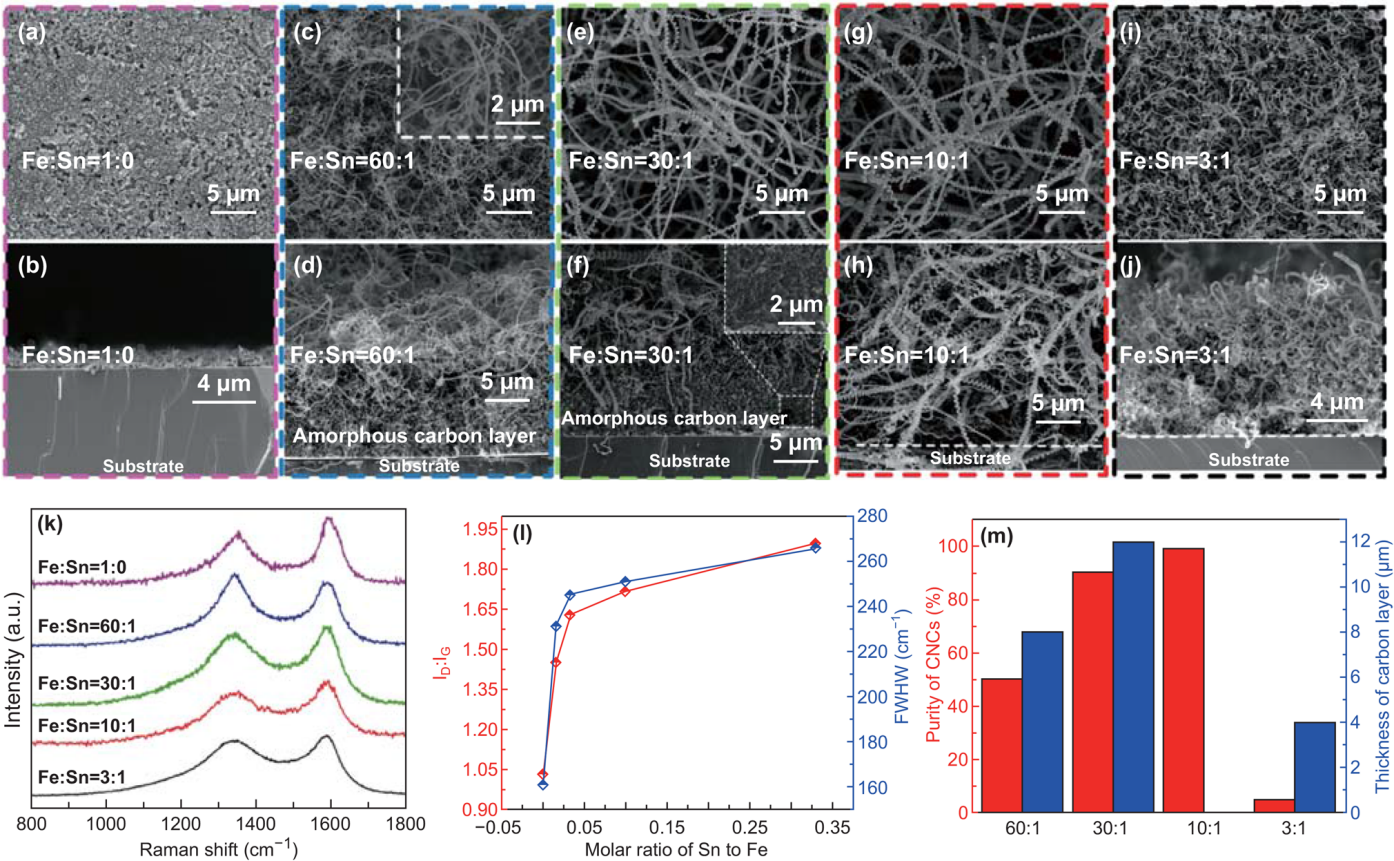
Top-view and cross-sectional SEM images of the carbon deposits prepared by the catalysts with different Fe/Sn molar ratios of **a, b** 1:0, **c, d** 60:1, **e, f** 30:1, **g, h** 10:1, and **i, j** 3:1. **k** Raman spectra and **l** the respective *I*_D_/*I*_G_ and FWHW values for the carbon deposits; **m** the thickness of carbon layer and purity of CNCs synthesized at different Fe/Sn molar ratios

In order to optimize the composition of Sn in catalyst, we prepared five kinds of nanoparticle catalysts with various molar ratios of Fe and Sn. Figure 1a–j shows a series of top-view and cross-sectional SEM images of carbon deposits on Si substrates using Fe/Sn catalysts with different molar ratios of 1:0, 60:1, 30:1, 10:1, and 3:1, respectively. It is found that with the change of Sn compositions in catalysts, the morphologies of carbon deposits are significantly different. As shown in Fig. 1a, b, the deposits are carbon nanoparticles when the catalyst does not contain Sn and no CNCs or carbon nanotubes are synthesized. With the increase in Sn content, CNCs with different morphologies are successfully synthesized as shown in Fig. 1c–h. Under the Fe/Sn molar ratio of 60:1 (as shown in Fig. 1c), the carbon deposits are spring- and twist-like CNCs with an average line diameter of approximately 160 nm. However, it cannot be ignored that the purity of CNCs is only about 50% and the by-product was identified clearly from Fig. 1d. CNCs with larger average line diameter and average coil diameter are successfully synthesized under the Fe/Sn molar ratios of 30:1 and 10:1 as shown in Fig. 1e and g, respectively. Nevertheless, a dense by-product layer between the base of the CNCs and substrate is observed in Fig. 1f. The enlarged image of the area indicated by the box in Fig. 1f shows the morphology of by-product layer, which is mainly composed of carbon-containing catalytic metal particles [42].

It is gratifying that under the Fe/Sn molar ratio of 10:1, as shown in Figs. 1h and S3, although some thin and irregular carbon nanowires are observed on the surface of substrate, the by-product layer has been eliminated completely and the CNCs with nearly 99% purity are obtained (Originated from ~ 211 CNCs and CNFs estimated by the top-view SEM images. Among them, there are 1 CNFs without spiral morphology, as shown in Fig. S3a. We also give the purity based on the section cross-sectional SEM image. As shown in Fig. S3b, a total number of 236 CNCs and 6 CNFs were identified). This purity is much higher than any of the reported values, suggesting that the catalyst having Fe/Sn molar ratio of 10:1 has high catalytic activity. In other words, the proportion of the “true” catalyst suitable for the growth of CNCs is greatly increased under this condition, and high-purity CNCs can be synthesized in large-scale by this kind of catalyst. When the Fe/Sn molar ratio of catalyst reaches 3:1 (Fig. 1i, j), the product becomes irregular and short CNFs. These results confirm that the content of Sn has important effects on the performance of catalyst, not only on the purity of CNCs, but also on the morphology of products. The carbon deposits prepared by catalysts with different molar ratios of Fe and Sn were studied by Raman spectroscopy at an excitation laser wavelength of 532 nm, as shown in Fig. 1k. There are two main peaks in the spectra: One is around 1322 cm^−1^, known as the D-band, which is originated from structural defects in carbon materials; the other one is around 1593 cm^−1^ named as G-band originated from graphite structure. The area ratio of the D-band and G-band is defined as *I*_D_/*I*_G_ which is used to evaluate the degree of graphitization. As shown in Fig. 1l, with the increase in Sn content in the catalyst, the *I*_D_/*I*_G_ ratio of the corresponding carbon deposit increases from 1.03 to 1.90, implying the increase in the amorphization of the carbon deposits. The full width at half maximum (FWHM) of the D-band also increases with the increase in Sn content, indicating that the unsaturated carbon atoms are more abundant for the carbon deposits prepared by catalysts with higher Sn/Fe ratio. The thickness of carbon layer and the purity of the CNCs prepared by catalysts with different molar ratios of Fe and Sn are presented in Fig. 1m. It is found that the purity of CNCs increases first and then decreases with the increase in Sn content in the catalyst, indicating that the appropriate ratio of Fe and Sn is needed for the high-efficiency growth of CNCs.

**Fig. 2:**
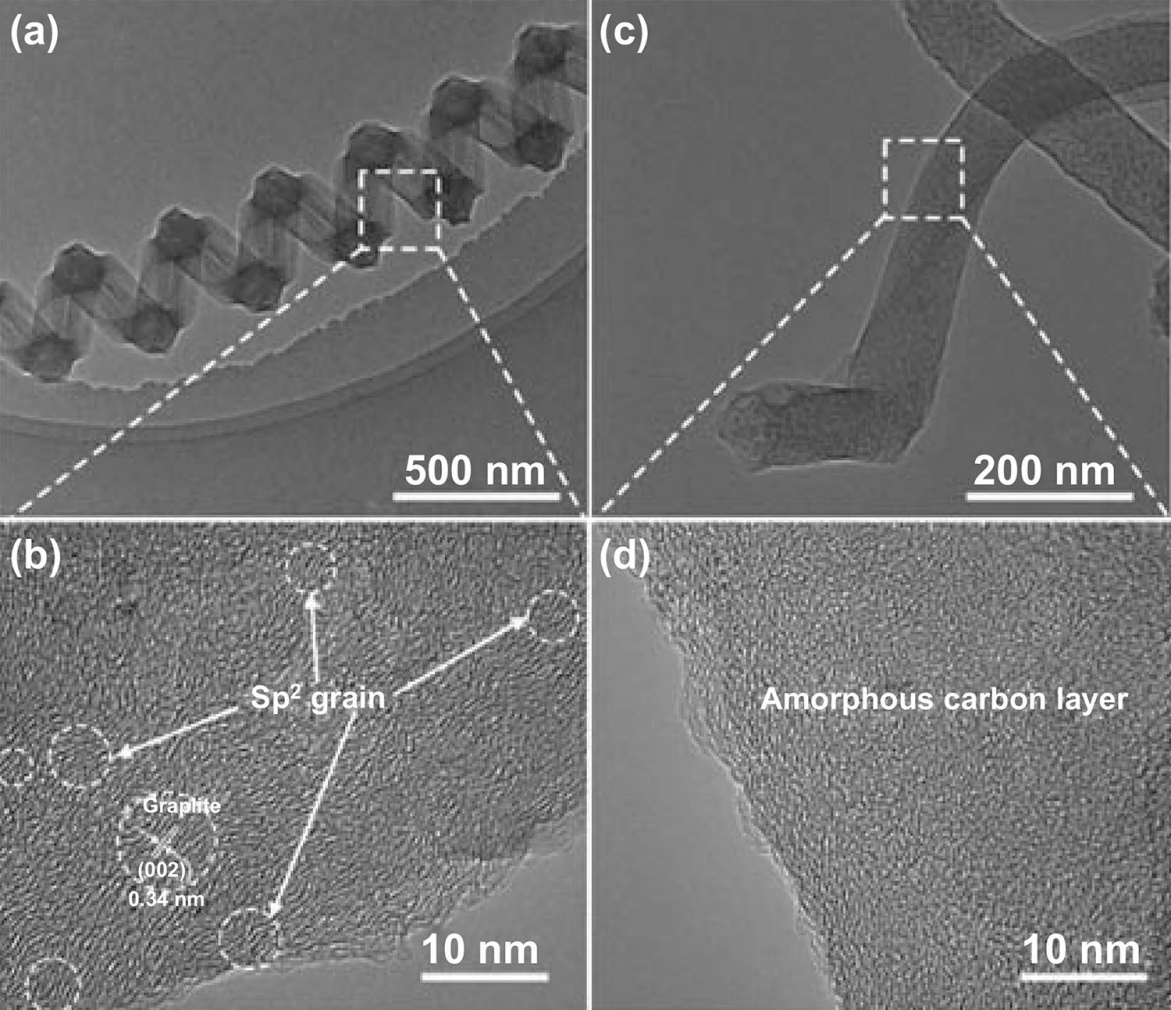
TEM and HRTEM images of a single CNC (**a, b**) and a single CNF (**c, d)**

Figure 2a is the TEM image of a single spring-like CNC with line diameter of 220 nm, coil diameter of 430 nm, and pitch of 500 nm, and its HRTEM image is shown in Fig. 2b. It is found that the lattice is partially ordered, indicating that many graphite grains (*sp*^2^ structured) are embedded in an amorphous network (*sp*^3^ structured), and the circles show that the *sp*^2^ grains have an average size of approximately 5 nm. The HRTEM image certifies that the CNCs synthesized under the Fe/Sn molar ratios of 10:1 have a polycrystalline-amorphous structure [47]. Figure 2c, d is the representative TEM and HRTEM images of a single CNF (from the deposit prepared by the catalyst with Fe/Sn molar ratio of 3:1) with a line diameter of approximately 120 nm. Unlike the structure of CNC, Fig. 2d shows that the lattice of CNF is completely disordered and the structure is amorphous.

#### Effects of Catalyst Densities

Our previous studies have shown that optimizing the film thickness or density of the catalyst significantly affects the morphology and purity of the synthesized carbon products [48, 49]. However, these are achieved by spin coating the catalyst precursor solution containing Fe and Sn or by adjusting the thicknesses of the Fe and Sn thin films in the magnetron sputtering process. Besides, the aggregation state of catalyst particles is also an important factor affecting the growth of CNCs. Therefore, we focus on the effect of changing the aggregation density of catalyst particles on the growth of CNCs. Figure S4 is a series of SEM images of catalyst aggregation prepared with different spin-coating times, and the samples are labeled as S_1_, S_3_, S_5_, S_10_, S_15_, and S_30_ corresponding to the coating times of 1, 3, 5, 10, 15, and 30, respectively. As shown in Fig. S4, the area density of the catalysts show a substantial increase from 7.1 × 10^8^ to 1.91 × 10^10^ cm^−2^.

**Fig. 3:**
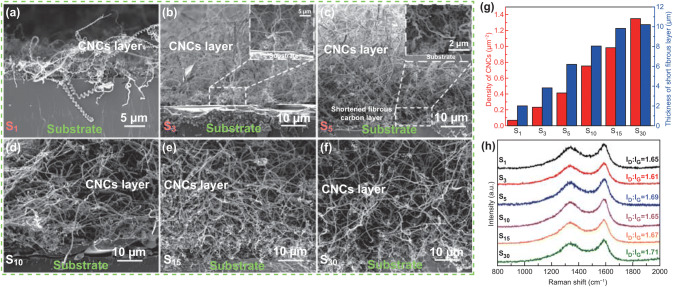
Cross-sectional SEM images of CNCs synthesized using Fe/Sn catalyst films with spin-coating times of **a** one, **b** three, **c** five, **d** ten, **e** fifteen, and **f** thirty times. **g** Effects of varying Fe/Sn catalyst density on the thickness of short fibrous carbon layers and density of CNCs. **h** Raman spectra of CNCs synthesized at different Fe/Sn catalyst films

Figure 3 shows the cross-sectional SEM images of CNCs synthesized using catalyst films prepared with different spin-coating times. It is observed that the CNCs synthesized by the spin-coated catalysts with different film thicknesses of S_1_ to S_30_ are basically the same in morphology and line diameter. Meanwhile, the growth density of CNCs increases with spin-coating times of catalyst. This may be due to the fact that the catalysts prepared are in the form of aggregates rather than monodisperse ones. It is observed from Fig. 3 that the carbon deposits have a bi-layer structure, i.e., a short fibrous carbon layer (inner part, confirmed by the enlarged images in Fig. 3b, c) and CNC layer (upper part). It is gratifying to find that the dense amorphous carbon layer disappears in all the samples of S_1_ to S_30_, which is quite different form the tri-layered structure (shown in Fig. 1f). The low-magnification SEM images of S_5_, S_10_, and S_30_ are shown in Fig. S5. These images show that high-purity CNCs can be synthesized efficiently under different catalyst aggregations, and no by-product carbon layer is produced. The low-magnification cross-sectional SEM image of the CNCs synthesized with coating times of fifteen is shown in Fig. S6a. It is observed that the whole product consists of CNCs, the height of the dense CNC layer reaches 80 μm, and many CNCs are higher than 100 μm. The enlarged images of Fig. S5a at different positions are shown in Fig. S6b–d. In each image, the uniform production of high-purity CNCs is well identified. In addition, as shown in Fig. 3g, the density of the CNCs increases from 0.07 to 1.35 μm^−2^ with increase in the density of the catalyst dispersions. Meanwhile, the thickness of short fibrous layer shows a similar increase trend. Furthermore, the intensity ratio of D to G peaks is shown in Fig. 3h. As the density of the catalyst increases, a very slight change of *I*_D_/*I*_G_ is observed. Therefore, we can confirm that the increase in catalyst density will not significantly affect the level of defects and disorder in CNCs. Hence, we believe that this facile strategy of preparing Fe/Sn catalyst particles to control the growth density of CNCs provides opportunities to boost their practical applications.

#### Yield of High-Purity CNCs

**Fig. 4:**
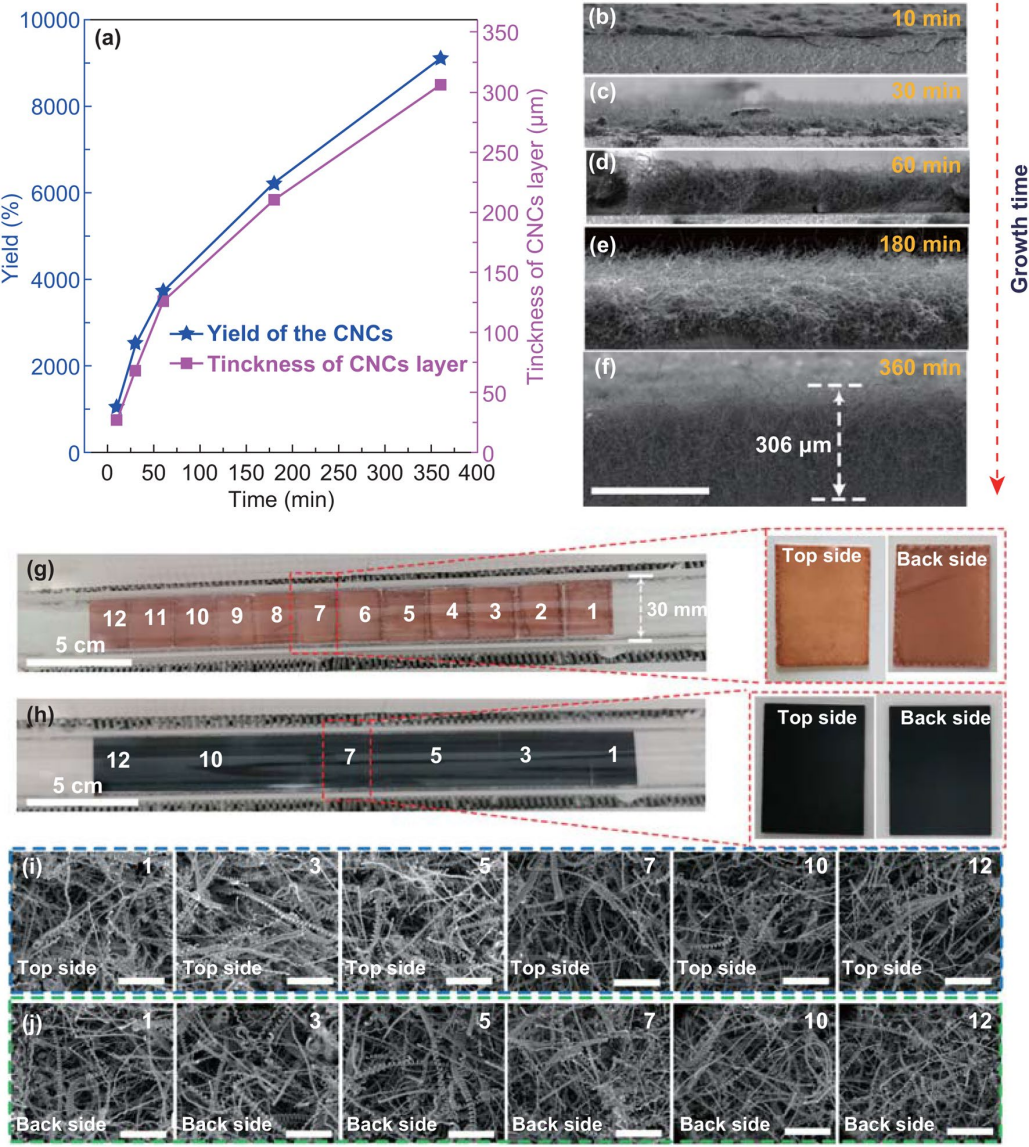
a Plots of yield and thickness of CNCs versus growth time; cross-sectional SEM images of CNCs grown for **b** 10, **c** 30, **d** 60, **e** 180, and **f** 360 min. Optical photographs of the substrates **g** before and **h** after the ‘scale-up’ CVD reaction. The scale bar for **b–f** is 300 μm. **i, j** Top and back sides SEM images of the carbon deposits prepared with different substrates of 1, 3, 5, 7, 10, and 12. The scale bar for **i** and **j** is 5 μm

Firstly, the dependence of yield and thickness of the CNCs on growth time was investigated carefully. As shown in Fig. 4a, with the increase in reaction time, the yield of CNCs increases apparently. It is noteworthy that the yield of the CNCs reaches 9,098% after a 6 h growth, which is much higher than those reported in the literature so far [23, 24, 29, 37]. This result suggests that the as-prepared catalyst has an excellent catalytic activity. Next, we measured the thicknesses of CNC layers at different growth times. Figure 4b–f shows cross-sectional SEM images of the CNCs grown for 10, 30, 60, 180, and 360 min, respectively. The relationship between the thicknesses of the CNC layers with growth time is plotted in Fig. 4a. It is found that the height of CNC layer continuously increased with growth time, and the maximum thickness of the CNC layer reaches 306 μm after the reaction for 6 h. This is the highest value compared with those reported recently [34–36, 40–42]. It is noted that both curves of the yield and thickness versus time are well matched, suggesting that the carbon deposits are almost CNCs. Furthermore, both of the curves rise with growth time without saturation, indicating that the catalyst remains high efficiency even after 6 h reaction.

Based on the results obtained, we performed a ‘scale-up’ experiment using 20 mg catalyst supported by 12 pieces of alumina substrates (size: 28 × 22 mm^2^, dip coating the catalyst on both sides of the substrate, labeled as 1 to 12, respectively.) in a quartz tube with inner diameter of 30 mm, as shown in Fig. 4g. After 1 h reaction, 729 mg carbon deposits were produced (as shown in Fig. 4h). The top and back sides of six substrates, labeled as 1, 3, 5, 7, 10, and 12, were examined by SEM carefully. Figure 4i, j shows a series of top and back sides SEM images of carbon deposits on substrates, and the results show that the CNCs with high purity are successfully synthesized in each position. This result suggests that nearly 150 cm^2^ area of high-purity CNCs can be obtained in a quartz tube with inner diameter of 30 mm. Since this process is simply operable and easily scalable, it is expected to be a promising method for large-scale commercial production of CNCs.

As listed in Table 1, we summarize various catalysts for the growth of CNCs reported in the literature. Among these reports, several catalysts achieved high-purity growth of CNCs, but their preparation processes are either complex/inefficient or use of chemical reagents containing noble metals, which are not suitable for mass synthesis. In addition, the high-purity CNCs/helical carbon nanotubes reported in Refs. [23] and [28] are short braided and do not have the morphologies of spring, which limits their applications in some fields. Furthermore, the yield of our CNCs in this work has reached a new record over the reported data. Therefore, it is clear that the as-prepared α-Fe_2_O_3_/SnO_2_ catalyst exhibits excellent performance with the characteristics of high catalytic efficiency, low-cost, and facile preparation.

**Table 1:** Comparison of various catalysts assisted growth of CNCs reported in the literature

No.	Catalysts	Preparation method	Purity^a^	Economy^b^	Thickness of carbon layer (μm)	Yield	Refs.
1	Ag/K	Thermal evaporation	Good	Fair	Several microns	–	[35]
2	Au/K	Thermal evaporation	Good	Fair	Several microns	–	[34]
3	Cu	ALD	Excellent	Fair	Few	–	[28]
4	BaSrTiO_3_/Sn	Mechanical mixing	Excellent	Fair	Few	–	[36]
5	Na/K	Solution method	Good	Good	–	1178%	[37]
6	Fe	Sol–Gel method	Excellent	Fair	Few	8078%	[23]
7	Fe/In/Sn	PVD	Good	Fair	–	–	[33]
8	Fe/Sn	Solution method	Good	Good	Tens of microns		[30]
9	Fe/Sn	PVD	Good	Fair	Tens of microns		[41]
10	Fe/Sn	Sol–Gel method	Good	Good	–	2510%	[29]
11	Fe/Sn	Solution method	Good	Good	Several of microns	–	[30]
12	Fe/Sn	PVD	Good	Fair	Several of microns	–	[42]
14	Fe	Sol–Gel method	Good	Good	–	7474%	[24]
15	Fe/Sn	Solvothermal method	Excellent	Good	Few	9098%	This work

^a^Excellent: The products are basically carbon nanocoils, and there is no by-product carbon layer; Good: The products are composed of carbon nanocoils and carbon layer

^B^Good: The equipment used is common, and the chemical reagents used are low cost. Fair: use of expensive equipment or chemical reagents containing noble metals

### Growth Mechanism of High-Purity CNCs

#### Analyses of the Catalyst

**Fig. 5:**
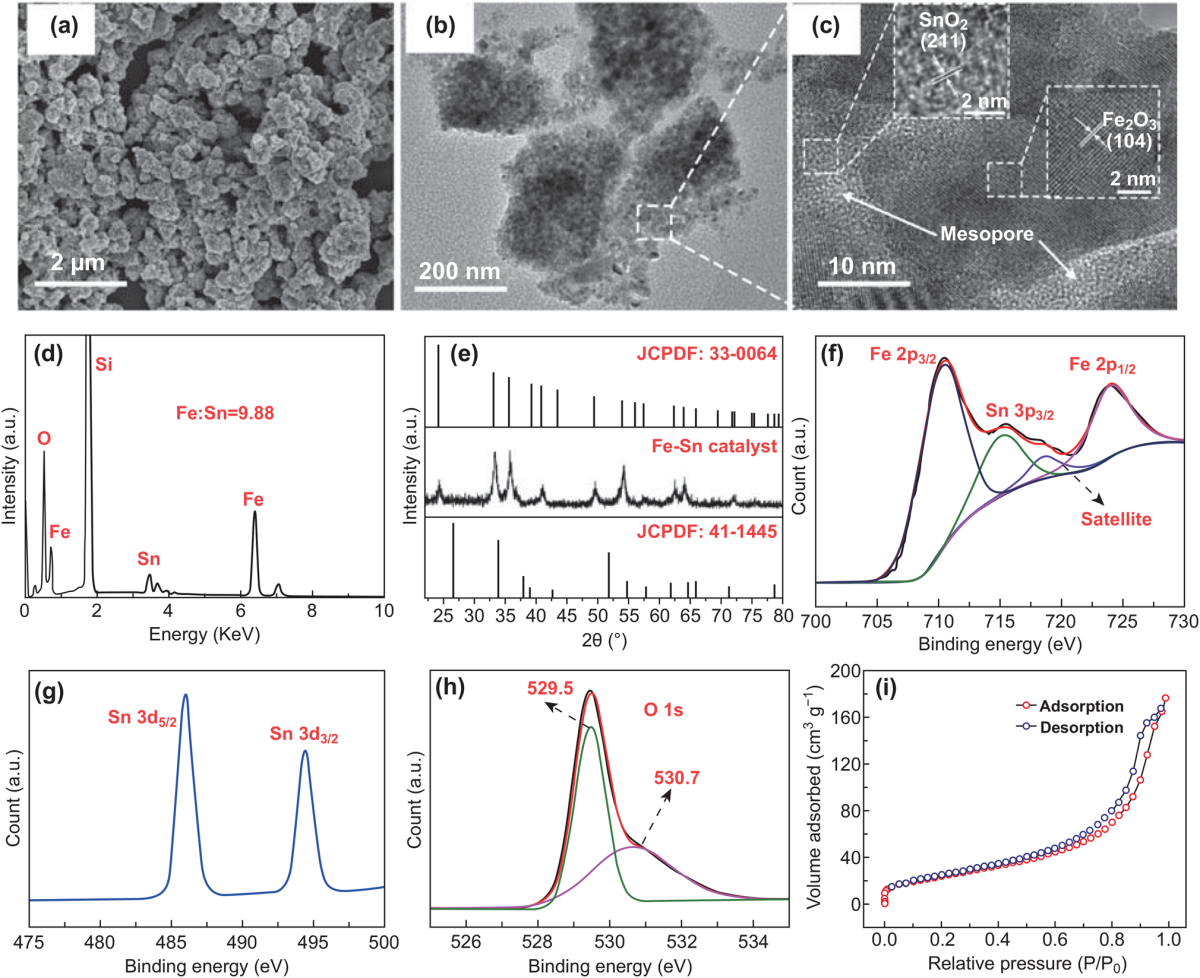
Structural and component analysis of the catalysts with Fe/Sn molar ratio of 10:1. **a** SEM, **b** TEM, and **c** HRTEM images, **d** EDX, and **e** XRD spectra, **f** Fe 2p and Sn 3p, **g** Sn 3d, and **h** O 1s XPS spectra, **i** N_2_ adsorption/desorption isotherm for the catalyst particles

In order to well understand the growth mechanism of high-purity CNCs from as-prepared catalyst, it is necessary to make clear the structure and composition of the catalyst particles. The catalysts prepared under the Fe/Sn molar ratio of 10:1 were analyzed in details. The SEM image of the catalyst film formed on Si substrate is shown in Fig. 5a. The catalysts are in the form of loose-porous nanoparticle aggregates and the nanoparticle with sizes distributed from 100 to 400 nm. It is observed from TEM image shown in Fig. 5b that the particle aggregates have an average size of approximately 200 nm, which consist of a large number of small and homogenous particles. HRTEM image of the designated area is shown in Fig. 5c. The lattice spacings of 0.176 and 0.270 nm correspond to the (211) plane of SnO_2_ (JCPDF No. 41-1445) and the (104) plane of α-Fe_2_O_3_ (JCPDF No. 33-0664), respectively. Furthermore, the mesoporous are also observed in the TEM and HRTEM images of the composite particles. Figure 5d shows the EDX spectrum of the catalyst particles. It is observed that the main components of the prepared catalysts are Fe, Sn, and O, with the molar ratio of Fe and Sn is 9.88:1 that is almost the same as the initial input molar ratio of Fe and Sn. The additional peak of silicon in the spectrum is derived from the supported Si substrate. XRD spectrum obtained from the catalysts is shown in Fig. 5e. All peaks in the spectrum can be well indexed to hematite (JCPDF No. 33-0664), indicating the formation of α-Fe_2_O_3_. No peak in the spectrum comes from SnO_2_ (JCPDF No. 41-1445), which is resulted from the small ratio of Sn in the catalysts. (XRD patterns and SEM images of the catalysts with different Fe/Sn molar ratios are given by Fig. S7.) The further evidence for the existence of Sn is the results of XPS shown in Fig. 5f–h. Figure 5f is the spectrum of Fe 2*p*, in which two peaks at 710.6 and 724.5 eV correspond to Fe 2*p*_3/2_ and Fe 2*p*_1/2_, respectively [50]. Figure 5g shows the spectrum of Sn 3*d*. There are two peaks at 486.2 and 494.6 eV corresponding to Sn 3*d*_5/2_ and Sn 3*d*_3/2_, respectively, which are originated from + 4 oxidation states of SnO_2_. Furthermore, the binding energy of 716.1 eV corresponding to Sn 3*p*_3/2_ is observed in Fig. 5f, which also supports the presence of SnO_2_ in the catalysts [51–53]. The O 1*s* spectrum is shown in Fig. 5h, with two peaks at 529.5 and 530.7 eV. The peak at 529.5 eV is derived from the defects and chemisorbed oxygen on the surface of Fe_2_O_3_, and the peak at 530.7 eV is attributed to the lattice oxygen in the α-Fe_2_O_3_/SnO_2_ composite [52]. A typical N_2_ adsorption–desorption isotherm of the catalysts is shown in Fig. 5i. The typical type IV isotherm in the relative pressure (P/P_0_) range of 0.45–0.90 indicates a well-developed meso-porosity in the catalyst nanoparticles, which is well in line with the SEM and HRTEM results. Furthermore, the BET surface area of the catalyst is measured to be 142.8 m^2^ g^−1^. Such a high value is due to the small particle size and porous structure of the catalyst. These porous aggregates have large contact area between supplied acetylene gas and particles, and thus improve the efficiency of CNC growth.

**Fig. 6:**
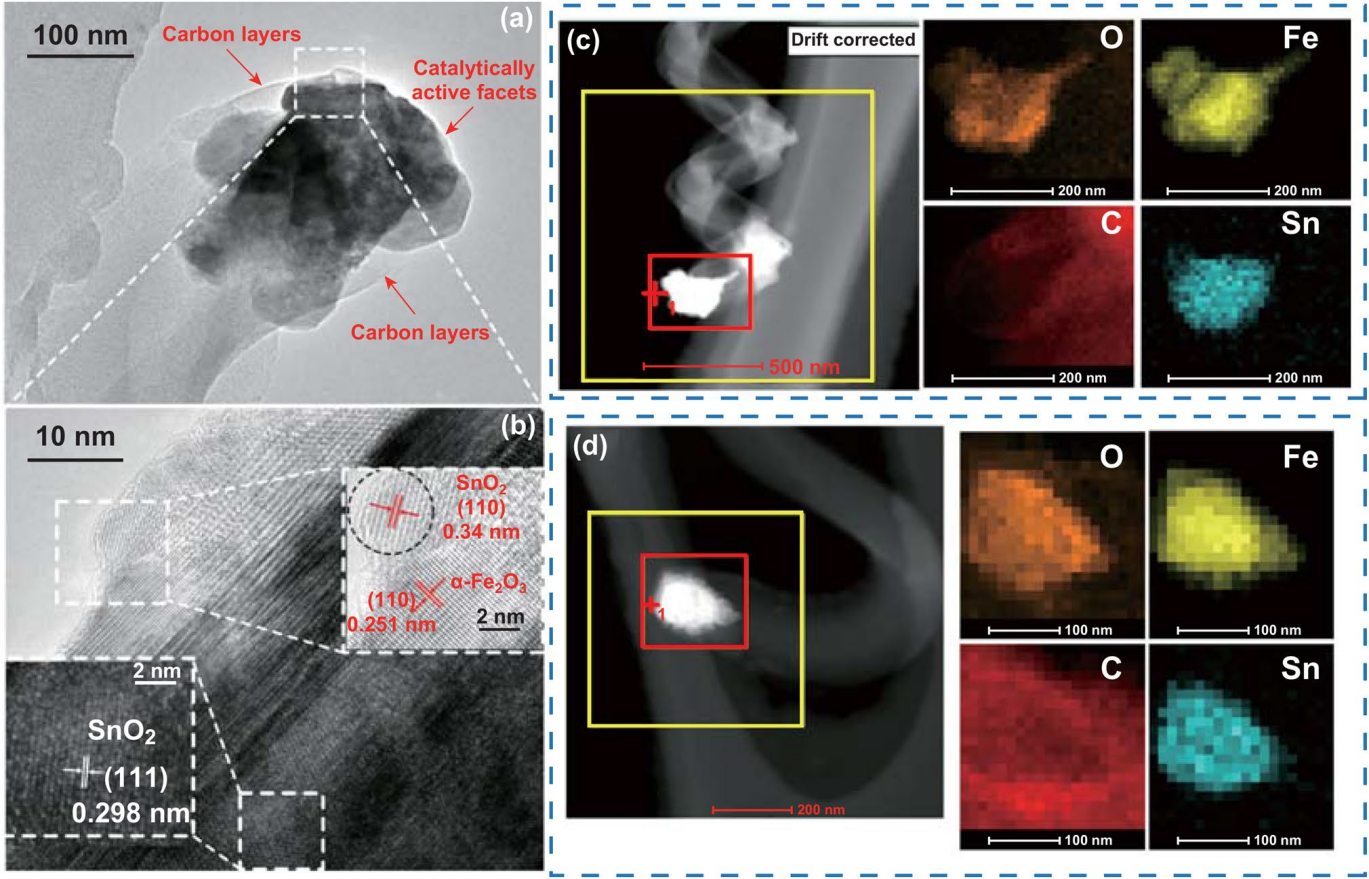
a TEM image of the tip of an as-grown CNC synthesized by the Fe/Sn catalyst with molar ratio of 10:1; **b** HRTEM image of the part indicated by a dashed box in **a**; **c**, **d** The elemental mapping of tip particles with Fe/Sn molar ratios of 10:1 and 3:1

Figure 6a shows a typical TEM image of the catalyst at the tip of an as-grown CNC synthesized by the α-Fe_2_O_3_/SnO_2_ catalyst under the molar ratio of 10:1. It is observed that the catalyst appears to have an irregular polyhedral shape and consists of two kinds of phases of with and without carbon layer. Figure 6b shows HRTEM image of the box area in the catalyst particle, which displays three typical lattice interlayer distances of 0.251, 0.298, and 0.34 nm and could be assigned to the (110) and (220) crystal planes of α-Fe_2_O_3_ and (110) planes of SnO_2_, respectively. Considering that Sn has a non-wetting interaction with graphite compared with that of the Fe [31], it is reasonable to believe that SnO_2_ attached to the surface of α-Fe_2_O_3_ most probably decreases the catalytic activity of this region. In order to solid our viewpoint, pure SnO_2_ was used as a catalyst for CVD reactions under the same conditions (710°, 235 sccm Ar, 25 sccm C_2_H_2_, 300 s), and the TEM and HRTEM results are given by Fig. S8. Only a small amount of amorphous carbon (Fig. S8b) were deposited on the surface of SnO_2_, suggesting that SnO_2_’s ability to decompose C_2_H_2_ gas and deposit carbon is insufficient. Furthermore, the elemental mapping of catalyst particles at the tips of two CNCs synthesized by the catalysts with Fe/Sn molar ratios of 10:1 and 3:1 is shown in Fig. 6c, d, respectively. It is observed from Fig. 6c that the distribution area of Sn is obviously smaller than that of Fe when the molar ratio of Fe and Sn is 10:1.

However, when the molar ratio is 3:1 (shown in Fig. 6d), the distribution area of Sn is basically equal to that of Fe and the grown fiber is no longer helical, but a curved and short CNF. Therefore, under our experimental conditions, the role played by SnO_2_ is summarized as follows: (I) The presence of SnO_2_ reduces the local catalytic activity of the α-Fe_2_O_3_ and prevents the catalyst from covered by the carbon. (II) The non-uniform distribution of SnO_2_ leads to the heterogeneous deactivation of the Fe_2_O_3_ catalyst, which leads to the anisotropy of the catalyst and promotes the helical nanocarbon growth.

#### Growth Mechanism of CNCs

**Fig. 7:**
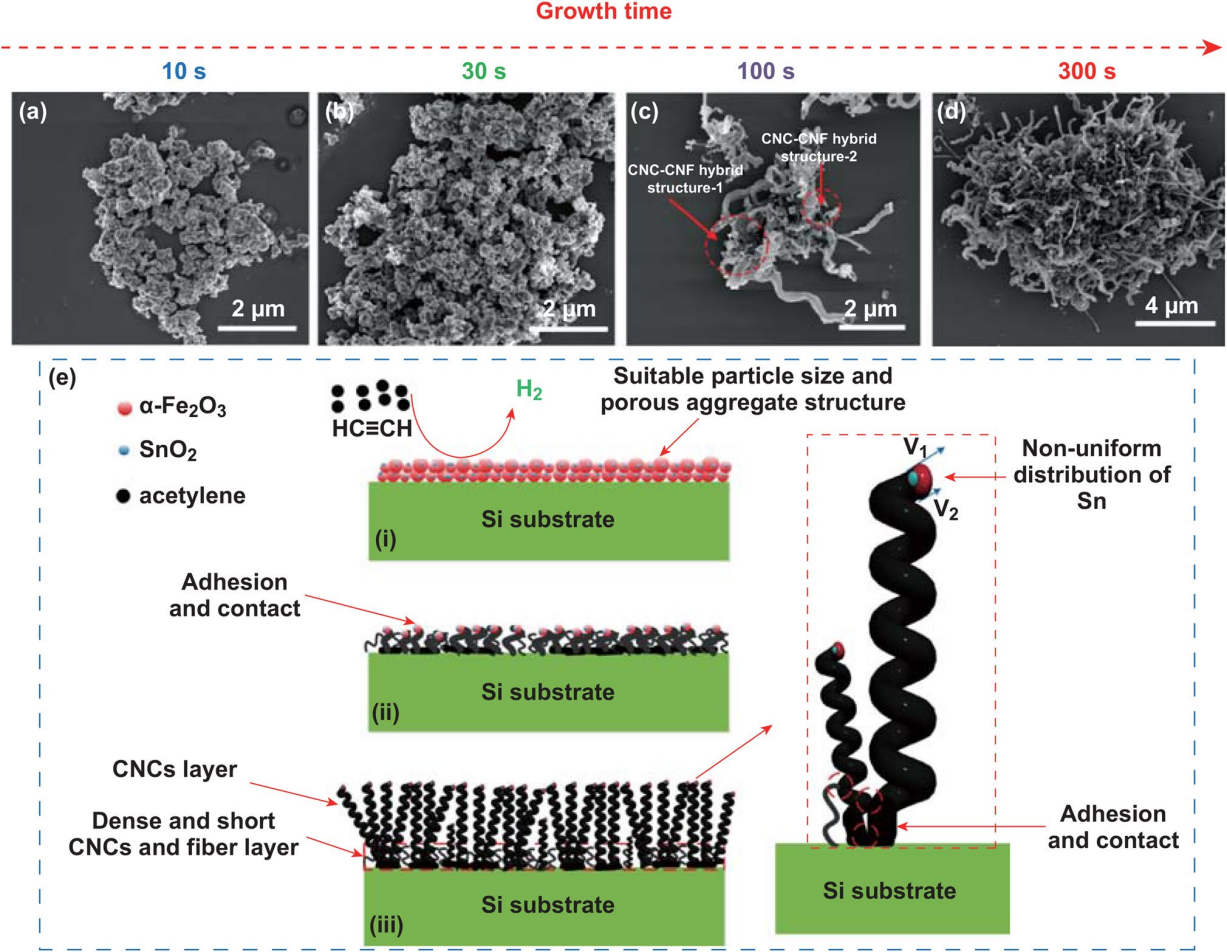
SEM images of the catalyst aggregates and deposits on the substrates after feeding C_2_H_2_ (4 sccm) at 710 °C for **a** 10 s, **b** 30 s, **c** 100 s, **d** 300 s. **e** Schematic of growth pathway of high-purity CNCs

Although several CNC growth mechanisms for Fe/Sn-based catalytic systems have been proposed, the origins and function of by-products have not been well understood yet. It is found that with increase in the spin-coating times (as mentioned in Sect. 3.2), the thickness of short fibrous layer gradually increases to a steady state. To investigate in details the origination of the short fibrous carbon layer, the product at the beginning of the CVD process was examined. Figure 7 shows a series of SEM images of catalyst aggregates and deposits after feeding C_2_H_2_ (4 sccm) at 710 °C for 10, 30, 100, and 300 s.

It is found that the morphology of catalyst aggregates changes with the C_2_H_2_ feeding time from 10 to 300 s. When the feeding time is increased from 10 to 30 s (Fig. 7a, b), a lot of fine particles are gradually formed on the surface of the catalyst aggregates. After feeding C_2_H_2_ for 100 s (Fig. 7c), some fibrous carbon and initial CNCs with a CNC–CNF hybrid structure have been synthesized. These results suggest that CNCs synthesized on the catalyst aggregates are likely to go through two stages: fibrous growth stage and spiral growth stage. It is accepted from mechanics point of view that the helical motion of a CNC generates a torsional moment on its base, which means that CNC itself requires a reaction force from the catalyst-carbon aggregate [54]. One reasonable explanation is that at the initial stage of CNC formation, the catalyst aggregate does not accumulate much carbon particles or fibrous carbon; therefore, it cannot provide enough solid base fixation for spiral growth. With the accumulation of carbon particles or fibrous carbon in the aggregate, the adhesion force between fiber and aggregate gradually increases. When the adhesion force can balance the torsional moment of its spiral growth, CNC begins to grow. It is also observed that the short fibrous layer is mainly formed at the root position of CNCs, which is considered to be derived from the catalyst particles not suitable for the growth of CNCs. With feeding C_2_H_2_ for 300 s, as shown in Fig. 7d, a large number of CNCs are grown from the surface of the catalyst aggregates, indicating that the catalyst particles in the form of aggregates are highly effective on the synthesis of CNCs. Thus, based on our experimental and analytic results, a growth pathway of CNCs is proposed, as shown by schematic diagrams in Fig. 7e. Herein, the classic vapor–liquid–solid model is used to explain the growth process of CNCs. The CVD growth process of CNCs is divided into three stages. At stage (i), the catalytically active phase of α-Fe_2_O_3_ particle assists the dissociation of C–H bonds and converts C_2_H_2_ into C atoms and H_2_, and then, these C atoms nucleate at precipitation phase and form carbon fiber, which is quite consistent with the experimental results observed in Figs. 6a, b and 7a. The presence of SnO_2_ reduces the local catalytic activity of the catalyst nanoparticle and prevents the catalyst covered by the carbon. Therefore, the amorphous carbon layer is greatly reduced and the catalyst efficiency is also significantly improved. It is worth noting that large specific surface area of the catalyst particles and the porous structure of the aggregates ensure their full contact with acetylene gas. Meanwhile, the porous structure of the catalyst aggregates provides necessary space for the growth of CNCs, which effectively improves the utilization of catalysts. At the next growth stage (ii), with increase in the amount of carbon deposition, a number of CNC, CNF, and CNC/CNF hybrid structures are grown from the catalyst aggregates, which are adhered or entangled with each other. It is reasonable to consider that the proper aggregation of catalyst particles is helpful for the root fixation during the growth of CNCs. Considering that the helical motion of a CNC during its growth generates a torsional moment on its base, therefore, the mutual adhesion and winding of CNC, CNF, and CNC/CNF hybrid provide the necessary rotary balancing moment for highly efficient growth of CNCs. At stage (iii), owning to the stable base fixation introduced by the adjacent CNCs and short fibrous carbon layer, as well as the non-uniform distribution of Sn on the tip catalyst particle induces the anisotropy of the catalyst, the CNC is grown with relatively uniform coil diameter and pitch.

### Fabrication of CNC Buckypaper

#### Electrical, Mechanical, and Electrochemical properties of CNC Buckypaper

**Fig. 8:**
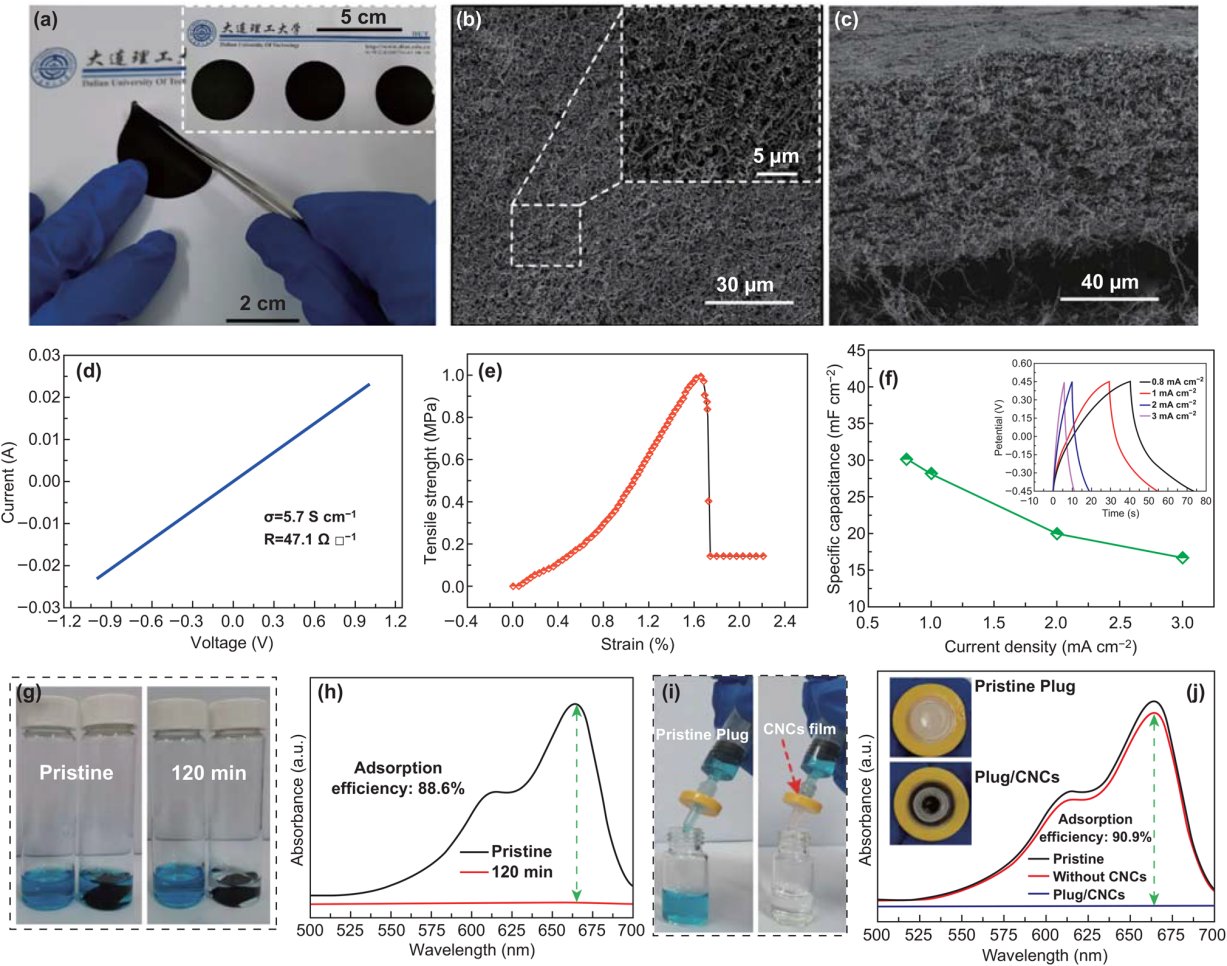
a CNC Buckypaper; **b** and **c** top-view and cross-sectional SEM images of CNC Buckypaper; **d** electrical, **e** mechanical, and **f** electrochemical properties of CNC Buckypaper. Insert of **e** galvanostatic charge/discharge measurement. **g** Photographs of a 10 ppm methylene blue solution (left) and the clear solution (right) obtained by soaking the CNC paper for 120 min. **h** UV–Vis spectra of pristine (10 ppm, black curve) and CNC paper-treated (red curve) methylene blue solution. **i** Photographs and **j** UV–Vis spectra of a 10 ppm methylene blue passing through the needle with pristine plug (left) or plug/CNC mix plug (right)

Due to the high-purity and large-scale preparation, a CNC Buckypaper has been successfully prepared. To the best of our knowledge, it is the first time that Buckypaper was prepared by CNCs. As shown in Fig. 8a–c, the diameter and thickness of the obtained CNC Buckypaper were about 35 mm and 80 μm, respectively. Due to the helical structure and long length of the synthesized CNCs, the CNC Buckypaper is flexible and has low density and rich porosity (bulk density: 0.075 g cm^−3^). To comprehensively understand the basic properties of CNC Buckypaper, the electrical, mechanical and electrochemical properties have been investigated. As shown in Fig. 8d, the conductivity and sheet resistance tested using four points probe were 5.7 S cm^−1^ and 47.1 Ω/□, respectively. As shown in Fig. 8e, the result of maximum strain range was 1.67%, which was little larger than that of carbon nanotube Buckypaper [55]. Meanwhile, the ultimate tensile strength reaches nearly 1 MPa. Electrochemical capacitive properties of the CNC Buckypaper were evaluated by cyclic voltammetry (CV) and galvanostatic charge/discharge (GCD) measurements. Figure S9a shows CV curves of the CNC paper at scan rates of 10, 20, 50, 100, and 200 mV s^−1^ with a potential window ranging from − 0.45 to 0.45 V in a 6 M KOH solution. The specific capacitances at various current densities are plotted in Fig. 8f and the highest area specific capacitance reaches 30.2 mF cm^−2^ (at the current density of 0.8 mA cm^−2^). These results suggested that CNC Buckypaper based capacitor shows a good capacitive behavior with the characteristic of double-layer capacitor.

#### CNC Buckypaper as Adsorbent for Removal of Methylene Blue

Based on the above results, we believe that the CNC Buckypaper has potential applications in many fields. Considering the advantages of its low density and rich porosity, it is a reasonable choice to utilize CNC Buckypaper as an adsorbent for the removal of pollutants from waste water. Figure 8g shows photographs of a 10 ppm methylene blue (5 mL) solution before (left) and after (right) soaking the CNC paper (2.25 cm^2^, 10.1 mg) for 120 min. UV–Vis spectra of methylene blue dye is shown in Fig. 8h. An adsorption efficiency of 88.6% is obtained, suggesting that the CNC Buckypaper has a good adsorption performance for methylene blue. Furthermore, a continuous-flow filtering experiment was performed to remove methylene blue dye in the solution. As shown in Fig. 8i, 10 mg of CNCs were packed into the filtration system (confirmed by insert of Fig. 8j), an aqueous solution of methylene blue dye (10 ppm) was pressed to pass through the packed CNC film at 298 K. The color disappearance clearly suggests that most of the methylene blue dye is adsorbed by the CNC membrane, and UV–Vis spectra of methylene blue dye confirms that the adsorption efficiency is 90.9%. Meanwhile, the adsorption capacity of CNCs was also be evaluated by UV–Vis spectra of methylene blue after adsorption at different time. As shown in Fig. S10, the adsorption capacity of methylene blue onto CNCs is 57.3 mg g^−1^, which is nearly twice of that for carbon nanotubes [56]. It is reasonable that the good adsorption ability of CNC originates from their relatively large specific surface area (131.2 m^2^ g^−1^, as shown in Fig. S11) and rough surface (confirmed by insert of Fig. S10b).

## Conclusions

CNCs with high purity of ~ 99% have been synthesized by using porous α-Fe_2_O_3_/SnO_2_ catalyst particles under Fe/Sn molar ratio of 10:1. Furthermore, the density of high-purity CNCs can be easily controlled by changing the density of the catalyst aggregates. The carbon deposit has little amorphous carbon layer, and the yield of the CNCs reaches 9098% in a 6 h reaction. Both the purity and yield of the CNCs are much higher than those reported in the literature. It is confirmed that the appropriate proportion of Fe and Sn, proper particle size distribution, and the loose-porous aggregates of the catalysts are the key points to the high-purity growth of the CNCs. Benefiting from the high-purity and efficient production, a CNC Buckypaper has been successfully prepared and the electrical, mechanical, and electrochemical properties were investigated comprehensively. Furthermore, the CNC Buckypaper was successfully utilized as an efficient adsorbent for the removal of methylene blue dye with an adsorption efficiency of 90.9%. We strongly believe that this work has a significant guiding importance in terms of efficient and large-quantity synthesis of high-purity CNCs at high yield. On the other hand, the fabrication of macroscopic CNC Buckypaper provides promising alternative for pollutant adsorption or other practical applications.

## Electronic supplementary material

Below is the link to the electronic supplementary material.

Supplementary material 1 (PDF 1230 kb)
