# Supplementary material for: Growth of Carbon Nanocoils by Porous α-Fe2O3/SnO2 Catalyst and Its Buckypaper for High Efficient Adsorption
Source: Nanomicro Lett. 2020 Jan 16;12:23. doi: 10.1007/s40820-019-0365-y (PMC7770930; doi:10.1007/s40820-019-0365-y)
Supplement: Supplementary file 1 — Supplementary material 1 (PDF 1230 kb) [file 40820_2019_365_MOESM1_ESM.pdf]

Supporting Information for

Growth of Carbon Nanocoils by Porous α-Fe_2O_3/SnO_2 Catalyst and Its Buckypaper for High Efficient Adsorption

Yongpeng Zhao[1, 2], Jianzhen Wang[1], Hui Huang[1], Tianze Cong[1], Shuaitao Yang[1], Huan Chen[2], Jiaqi Qin[3], Muhammad Usman[1, 4], Zeng Fan[1] , Lujun Pan[1, *]

[1]School of Physics, Dalian University of Technology, Dalian, Liaoning 116024, People's Republic of China

[2]School of Microelectronics, Dalian University of Technology, Dalian, Liaoning 116024, People's Republic of China

[3]State Key Laboratory of Fine Chemicals, School of Chemical Engineering, Dalian University of Technology, Dalian, Liaoning 116024, People's Republic of China

[4]Department of Physics, Khawaja Fareed University of Engineering and Information Technology, Rahim Yar Khan, 64200, Pakistan

*Corresponding author. E-mail: lpan@dlut.edu.cn (L. Pan)

S1 Synthesis of High Purity Carbon Nanocoils

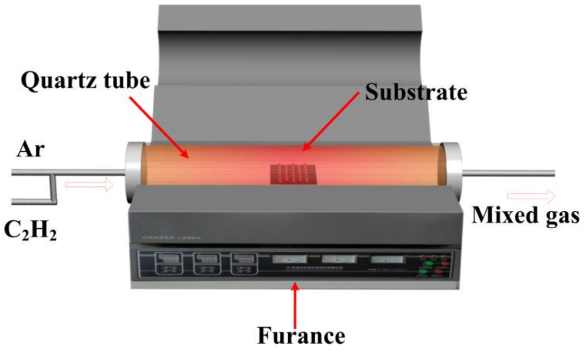

Fig. S1 The schematic of CVD apparatus with position of substrate

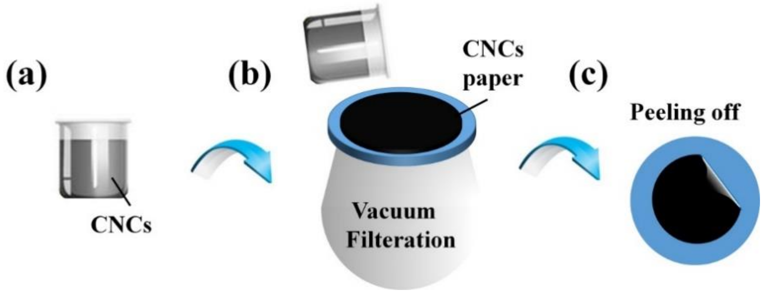

Fig. S2 The schematic of fabrication process of CNC buckypaper

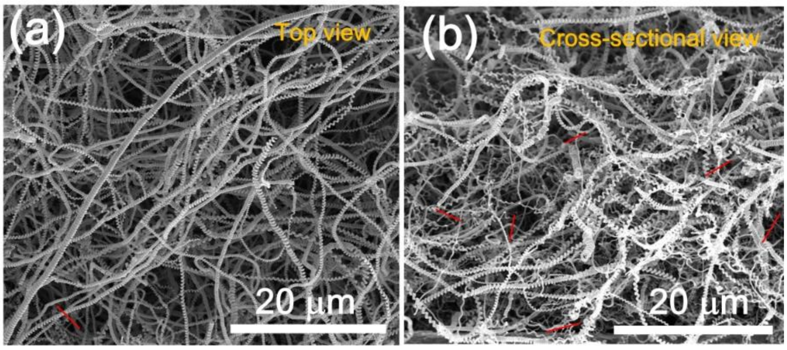

Fig. S3 Top view and cross-sectional SEM images of the CNCs prepared by the catalysts with Fe/Sn molar ratios of 10:1

In this paper, the purity of CNC is defined by Eq. S1. Therefore, the key to assessing purity is to determine the number of CNCs and CNFs. In this paper, CNFs with spring-like, twist-like and braided-like structures were defined as CNCs. It is worth noting that some CNCs do not have helical morphology at their initial growth stage, and this kind of CNFs with CNC-CNF hybird structure only in their roots are also classified as CNCs On the other hand, a large number of literatures have reported that the purity of CNC can be evaluated by top view SEM [S1-S4]. Therefore, based on this evaluation standard, a total number of 211 CNCs and CNFs was identified (Fig. S3a). Among them, there are 1 CNFs without spiral morphology. As a result, according to equation (1), the purity of CNC is 99.7%. In addition, we also give the purity based on the section cross-sectional SEM image. As shown in Fig. S3b, a total number of 235 CNCs and 6 CNFs were identified. Therefore, based on such evaluation criteria, the synthetic purity of CNC is 97.5%.

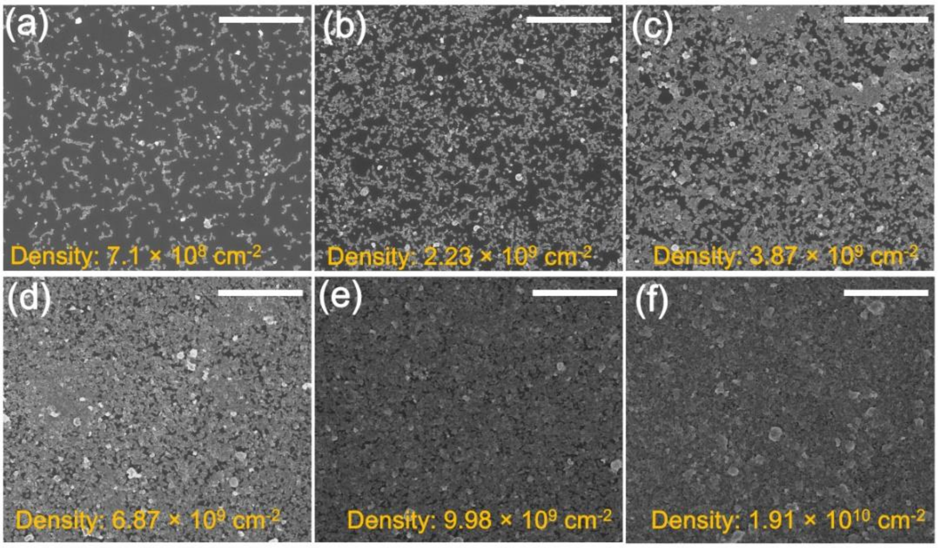

Fig. S4 SEM images of Fe/Sn catalyst films with spin-coating times of (**a**) one, (**b**) three, (**c**) five, (**d**) ten, (**e**) fifteen, and (**f**) thirty times, respectively. The scale bar of **(a-f)** is 4 μm

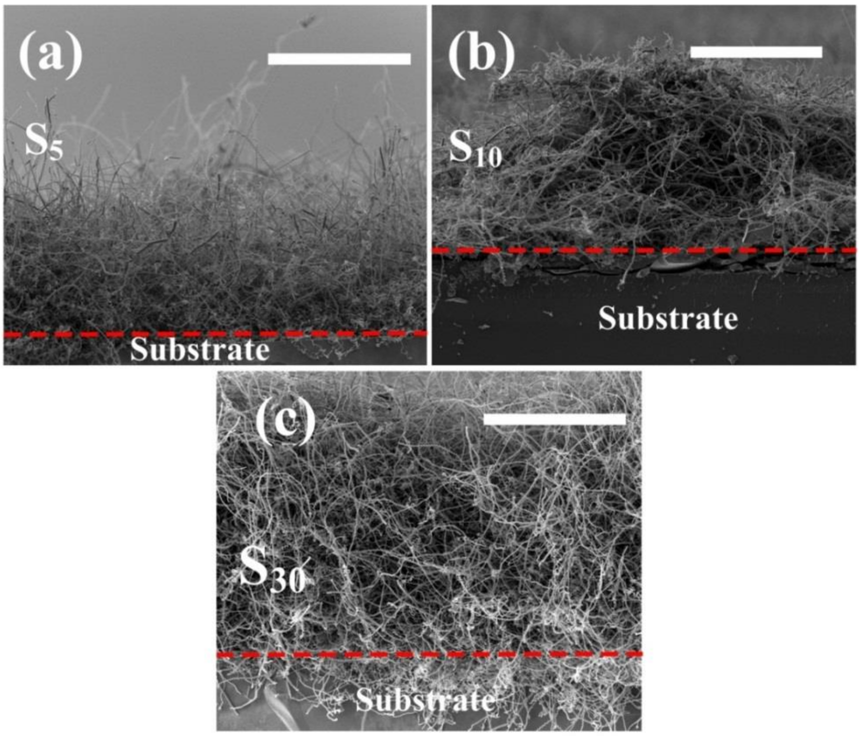

Fig. S5 Low magnification cross-sectional SEM images of carbon nanocoils synthesized using Fe-Sn catalyst films with different spin-coating times of (**a**) fifth, (**b**) ten, and (**c**) thirty. The scale bar of (**a-c**) is 40 μm

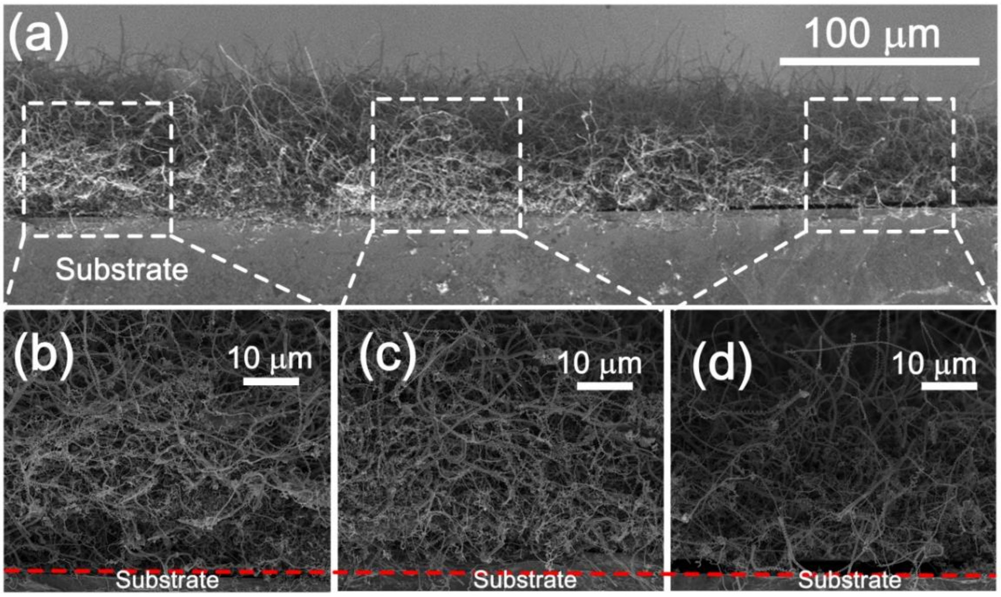

Fig. S6 Cross-sectional SEM image of CNCs synthesized with spin-coating times of fifteen: (**a**) low magnification image and images of the CNCs on substrate at different positions: (**b**) left side, (**c**) middle, and (**d**) right side

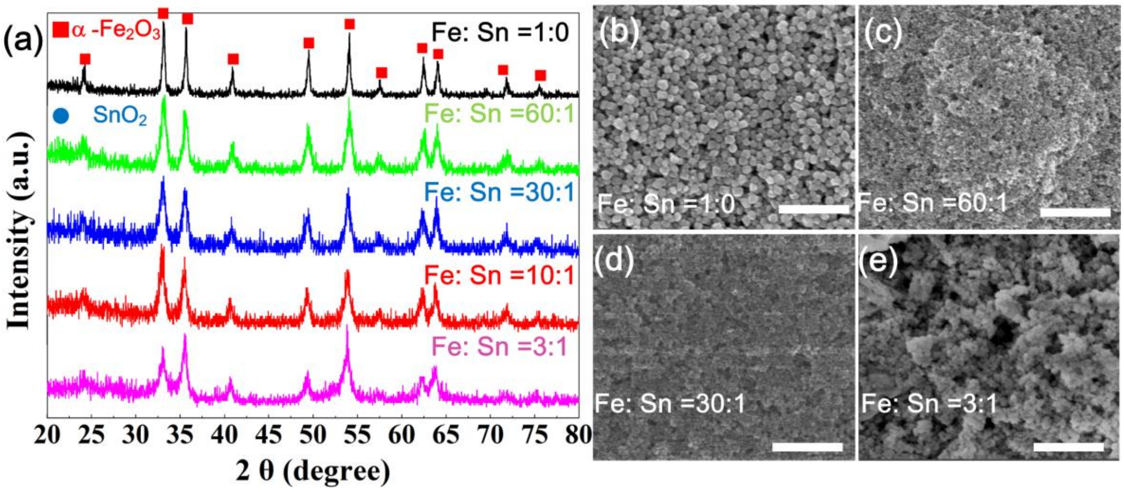

Fig. S7 (**a**) XRD spectra of the catalysts with different Fe/Sn molar ratios. SEM images of the catalysts with different Fe/Sn molar ratios of (**b**) 1:0, (**c**) 60:1, (**d**) 30:1, and (**e**) 3:1, respectively. The scale bar of (**b-e**) is 500 nm

As shown in Fig. S7a, all peaks in the spectrum are well indexed to α-Fe_2O_3 and no peak in the spectrum comes from SnO_2. These results suggest that the SnO_2 in the catalyst is amorphous. The morphology evolution of catalysts with different Fe/Sn molar ratios is shown by SEM images in Fig. S7b-e. Figure 7b shows the morphology of catalysts under the Fe/Sn molar ratio of 1:0. It is clearly observed that uniform polyhedral nanoparticles with average size of 70 nm were synthesized successfully. The corresponding XRD pattern (**Fig.** S7a, black curve) shows sharp and strong peaks, indicating the high crystallinity of -Fe_2O_3. However, with the increase of Sn content, the size of catalyst nanoparticles decreases gradually, and the agglomeration of catalyst nanoparticles is more serious. Furthermore, the broadening of XRD peaks also indicates that the size of the catalyst particles decreases with the increase of Sn.

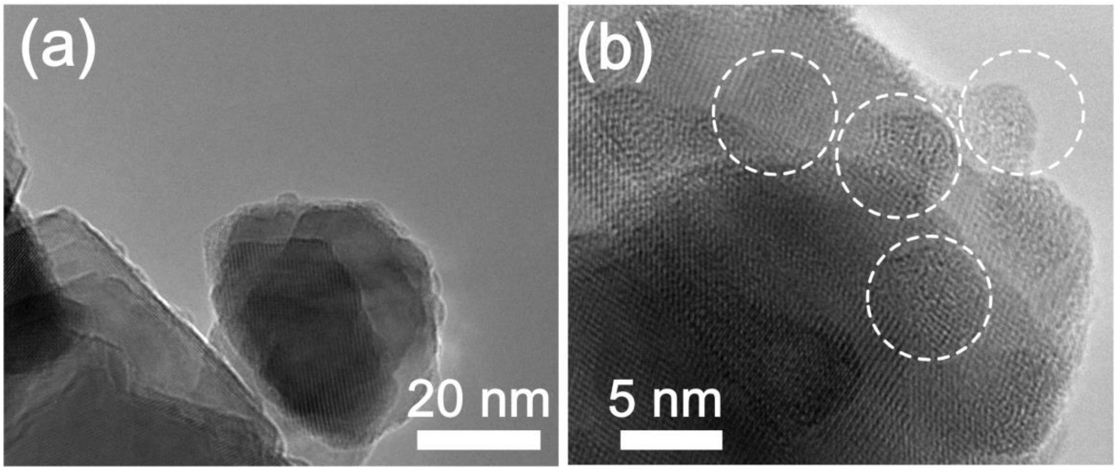

Fig. S8 (**a**) TEM and (**b**) HRTEM images of the pure SnO_2 aggregates after feeding C_2H_2 (10 sccm) at 710 °C for 300 s

S2 Electrochemical Methods

The electrochemical measurements of the CNC buckypaper were carried out using a CHI660E electrochemical workstation with a three-electrode system in 6 M KOH electrolyte. The counter electrode was platinum sheet and the reference electrode was constructed using Ag/AgCl electrodes. The area specific capacitance of the CNC Buckypaper is calculated using Eq. S1:

$$C_m = (I\Delta t)/(m\Delta V) \qquad (S1)$$

where C_m, I, Δt, m, and ΔV represent the aera specific capacitance of the CNC paper, the discharge current (A), the discharge time (s), the area of electrode materials (g) and the device voltage (V) after the IR drop, respectively.

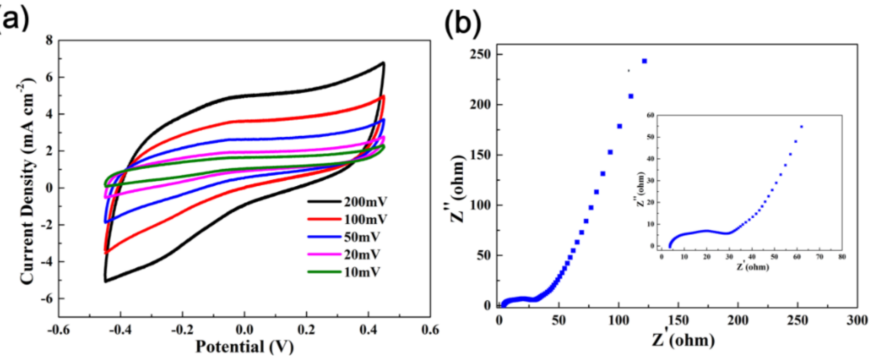

Fig. S9 (**a**) CV curves at varied sweep rates increasing from 10 to 200 mV s^{-1}. (**b**) Nyquist plots of the CNC buckypaper electrode measured under the potential with amplitude of 5 mV over the frequency range from 100000 to 0.1Hz

S3 Methyl Blue Dye Absorption

The pristine solution of methylene blue (MB) dye was prepared by dissolving certain amount of MB dye into the DI water to get 10 ppm (10 mg L^{-1}) of MB solution. The concentration of MB was monitored by using a UV–vis spectrophotometer at its characteristic wavelength (664 nm). In a typical experiment, 5 mg purified CNCs and 30 mL of MB solution with initial concentrations (C_0) of 10 mg L^{-1}. The temperature was held at 298K and the pH was fixed at 7. The adsorption capacity of MB at equilibrium q_e (mg g^{-1}) was calculated from Eq. S2:

$$q_e = (C_0 - C)\, V/m \qquad (S2)$$

where C_0 and C are the initial and equilibrium concentrations of MB dye (mg L^{-1}) in solution, respectively; m is the weight of the CNCs (g) and V is the volume of the solution.

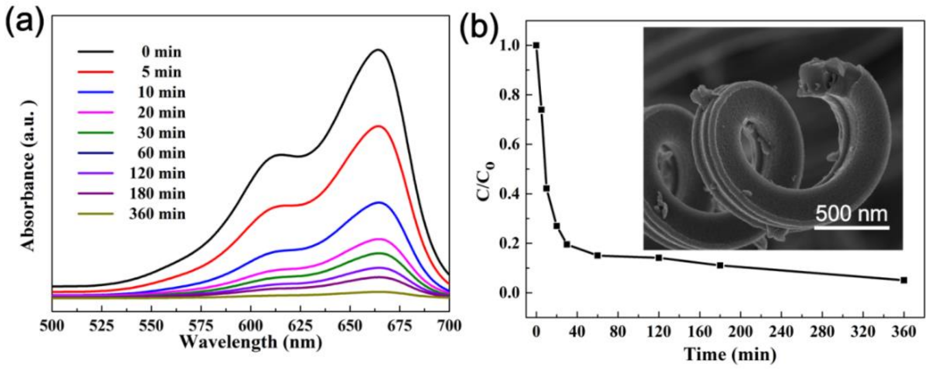

Fig. S10 (**a**) UV-Vis spectra of MB dye after adsorption at different time intervals. (**b**) Adsorption isotherms of methylene blue at 298K

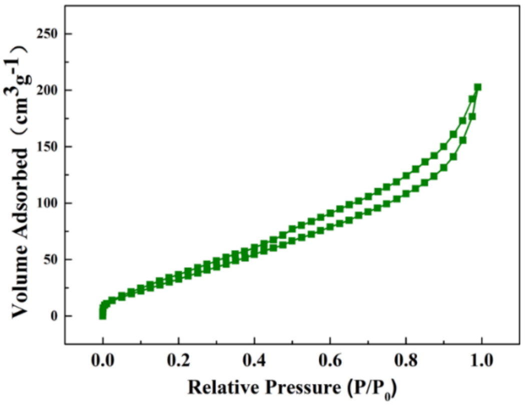

Fig. S11 N_2 adsorption/desorption isotherm for the CNCs

Supplementary References

[S1] F. Meng, Y. Wang, Q. Wang et al., High-purity helical carbon nanotubes by trace-water-assisted chemical vapor deposition: Large-scale synthesis and growth mechanism. Nano Res. **11**(6), 3327-3339 (2018). https://doi.org/10.1007/s12274-017-1897-4

[S2] G. Wang, G. Ran, G. Wan, P. Yang, Z. Gao, S. Lin, C. Fu, Y. Qin. Size-selective catalytic growth of nearly 100% pure carbon nanocoils with copper nanoparticles produced by atomic layer deposition. ACS Nano **8**(5), 5330-5338 (2014). https://doi.org/10.1021/nn501709h

[S3] D. Li, L. Pan, J. Qian, D. Liu. Highly efficient synthesis of carbon nanocoils by catalyst particles prepared by a sol–gel method. Carbon **48**(1), 170-175 (2010). https://doi.org/10.1016/j.carbon.2009.08.045

[S4] X. Jian, M. Jiang, Z. Zhou, M. Yang, J. Lu, S. Hu, D. Hui, Preparation of high purity helical carbon nanofibers by the catalytic decomposition of acetylene and their growth mechanism. Carbon **48**(15), 4535-4541 (2010). https://doi.org/10.1016/j.carbon.2010.08.035
